# Identification and characterization of CircRNAs involved in the regulation of wheat root length

**DOI:** 10.1186/s40659-019-0228-5

**Published:** 2019-04-04

**Authors:** Yanhua Xu, Yongzhe Ren, Tongbao Lin, Dangqun Cui

**Affiliations:** 1 College of Agronomy, Henan Agricultural University, Zhengzhou, 450002 China; 2 State Key Laboratory of Wheat and Maize Crop Science, Henan Agricultural University, Zhengzhou, 450002 China; 3 Collaborative Innovation Center of Henan Grain Crops, Henan Agricultural University, Zhengzhou, 450002 China; 4 College of life science, Shangqiu Normal University, Shangqiu, 476000 China

**Keywords:** *Triticum aestivum* L., Root length, CircRNAs, Regulation

## Abstract

**Background:**

Recent studies indicate that circular RNAs (circRNAs) may play important roles in the regulation of plant growth and development. Plant roots are the main organs of nutrient and water uptake. However, whether circRNAs involved in the regulation of plant root growth remains to be elucidated.

**Methods:**

LH9, XN979 and YN29 are three Chinese wheat varieties with contrasting root lengths. Here, the root circRNA expression profiles of LH9, XN979 and YN29 were examined by using high-throughput sequencing technology.

**Results:**

Thirty-three and twenty-two differentially expressed circRNAs (DECs) were identified in the YN29-LH9 comparison and YN29-XN979 comparison, respectively. Among them, ten DECs coexisted in both comparisons. As the roots of both LH9 and XN979 were significantly larger and deeper than YN29, the ten DECs coexisting in the two comparisons were highly likely to be involved in the regulation of wheat root length. Moreover, three of the ten DECs have potential miRNA binding sites. Real-time PCR analysis showed that the expression levels of the potential binding miRNAs exhibited significant differences between the long root plants and the short root plants.

**Conclusions:**

The expression levels of some circRNAs exhibited significant differences in wheat varieties with contrasting root phenotypes. Ten DECs involved in the regulation of wheat root length were successfully identified in which three of them have potential miRNAs binding sites. The expression levels of putative circRNA-binding miRNAs were correlated with their corresponding circRNAs. Our results provide new clues for studying the potential roles of circRNAs in the regulation of wheat root length.

**Electronic supplementary material:**

The online version of this article (10.1186/s40659-019-0228-5) contains supplementary material, which is available to authorized users.

## Background

Plant roots are the main organs of nutrient and water uptake from soil. Genetic improvement of root traits is of vital importance for improving crop nutrient and water use efficiencies [1–5]. Understanding the molecular mechanisms controlling plant root growth and development is beneficial to molecular breeding aimed at improving root traits and resource utilization efficiency of crops. Previous studies have shown that plant root growth and development are controlled by phytohormones and sustained by the root apical meristem (RAM) [6–12]. Besides, redox regulation and the balance of ROS also have important roles in maintaining RAM activity [13–15]. Therefore, the growth and development of plant roots are regulated by complex hormone signals and other related pathways.

CircRNAs are endogenous non-coding RNAs produced by back-splicing of pre-mRNA [16]. The 5′ and 3′ ends of the circRNA are linked together to form a covalent closed loop structure [17]. Currently, circRNAs have been widely studied in animals [18–21]. However, the role of circRNA in plants has not attracted enough attention [22]. Until recent years, several literatures reported circRNAs identified in plants such as *Arabidopsis* [23–27], soybean [28, 29], rice [25, 30], maize [31–33], tomato [34, 35], barley [36], tea [37], cotton [38, 39] and wheat [40, 41]. For example, Ye et al. performed genome-wide identification of circRNAs in rice and *Arabidopsis* using available public RNA-Seq data and identified 12,037 and 6012 circRNAs, respectively. Moreover, the parent genes of over 700 exonic circRNAs were orthologues between rice and *Arabidopsis*, suggesting the conservation of circRNAs in plants [25]. Chen et al. performed circRNA-Seq on maize seedling leaves and uncovered 2804 circRNAs. They found that sequences related to LINE1-like elements (LLEs) and their reverse complementary pairs (LLERCPs) are significantly enriched in the flanking regions of circRNAs [31]. Furthermore, genes with LLERCP-mediated circRNAs are enriched among loci that are associated with phenotypic variation. Therefore, circRNAs are likely to be involved in the modulation of phenotypic variation by LLERCPs [31]. Moreover, studies showed that circRNAs may also play roles in response to Verticillium wilt in cotton [39], maize iranian mosaic virus infection in maize [32], TYLCV infection in tomato [35], drought stress and nitrogen deficiency in wheat [38, 41]. These findings indicate that circRNAs are present in different plant species and may play important roles in the regulation of growth and development, and stress response.

Wheat (*Triticum aestivum* L.) is one of the most important food crops in the world. In wheat, circRNAs have been shown to be involved in response to drought stress. Wang et al. isolated 88 circRNAs and found that 62 circRNAs were differentially expressed under drought stress conditions compared with control [38]. In a recent study, Ren et al. identified six circRNAs involved in the common response to nitrogen deficiency stress and 23 circRNAs involved in the regulation of low nitrogen-promoted root growth in wheat [41]. These studies show that circRNAs may play important roles in responding to abiotic stresses. However, it is unclear whether circRNAs are involved in the regulation of wheat root growth under normal growth conditions. To explore this question, the root circRNA expression profiles of three wheat varieties (including two long root varieties and one short root variety) were obtained using high-throughput sequencing technology. Differentially expressed circRNAs (DECs) were identified and further validated using real-time PCR technology. Target miRNAs of the DECs were predicted and the expression levels of these miRNAs in the roots of LH9, XN979 and YN29 were also examined. This is the first report on the identification of differentially expressed circRNAs in wheat varieties with contrasting root length. The results are helpful for further investigate of the potential roles of circRNAs in regulating wheat root length.

## Methods

### Plant materials

XN979, LH9 and YN29 are three Chinese wheat varieties. The roots of LH9 and XN979 are significantly longer than those of YN29. Here, XN979, LH9 and YN29 were selected as materials to identify circRNAs involved in the regulation of wheat root length.

### Plant growth conditions and evaluation of root phenotype

Seed sterilization, germination and the growth conditions of wheat plants were conducted as previously described by Ren et al. [42]. Plants were randomly placed and grown in a greenhouse with six replications each. The maximum root length (MRL) and total root length (TRL) of XN979, LH9 and YN29 were analyzed by using WinRHIZO software (Regent Instruments, Canada) after 15 days of transferring. The developmental stages of wheat plants were Zadoks growth scale 13 [43]. The roots of XN979, LH9 and YN29 were fast-frozen using liquid nitrogen for RNA extraction.

### Libraries construction and sequencing

Root total RNA was extracted using Trizol reagent following the manufacturer’s procedure. The concentration and purity of total RNA were measured by NanoDrop ND-1000 spectrophotometer (NanoDrop Technologies, Wilmington, DE, USA). Approximately 10 µg total RNA was used to deplete the ribosomal RNA (rRNA) following the instructions of the Epicentre Ribo-Zero Gold Kit (Illumina, San Diego, USA). The remaining RNAs were used as templates for the construction of cDNA libraries following the instructions of the RNA-Seq sample preparation kit (Illumina, San Diego, USA) [41]. Three biological replicates were analyzed. The samples were named as XN979-1, -2, -3; LH9-1, -2, -3; YN29-1, -2, -3, respectively. Paired-end sequencing was performed on an Illumina Hiseq 2500 platform (Hangzhou Shangyi biotechnology company, Hangzhou, China).

### Identification of circRNAs

The obtained clean reads were tempted to align with wheat reference genome (*Triticum aestivum* TGACv1.0) using the bowtie2 (bowtie2-2.2.2) alignment method. The reads of the linear RNA can be mapped appropriately to wheat reference genome, while the reads at the loop forming junctions of circRNAs cannot be directly aligned to the wheat reference genome [41]. Then the find_circ software was employed to detect head-to-tail splicing (back-spliced) of the remaining unmapped RNA-seq reads (default setting). The detected back-spliced reads were further filtered to predict circRNAs following the recommended setting rules (GU/AG appears on both sides of the splice site, clear breakpoint can be detected; ≤ 2 mismatches; The length of the circRNA junctions ≤ 100 kb) [41, 44].

### Differential expression analysis of circRNAs

Differential expression analysis of circRNAs between different varieties was performed using the DEseq R package [41]. Only the circRNAs with p values ≤ 0.05 and |log2 (foldchange)| ≥ 1 were defined as DECs [45, 46]. psRNATarget software was used to predict circRNA-miRNA interactions of the DECs [47].

### Real-time PCR analysis

To confirm and quantify the predicted circRNAs, divergent primers of five randomly selected DECs were designed based on the flanking sequences of the head-to-tail splicing site of each circRNA (Additional file 1: Table S1). The cDNA samples were used as templates and mixed with primers and SYBR Green PCR Real Master Mix (Tiangen, China) for real-time PCR analyses. In the quantitative analysis of selected miRNAs, miRcute Plus miRNA First-Strand cDNA Synthesis Kit (Tiangen, China) was used for cDNA synthesis and miRcute Plus miRNA qPCR Detection Kit (Tiangen) was used for quantitative analysis according to the kit instructions. The reverse primers were provided in the miRcute Plus miRNA qPCR Detection Kit and the forward ones were designed according to the instructions of the kit (Additional file 1: Table S1). Real-time PCRs were performed on a Thermal Cycler CFX96 Real-Time System (Bio-Rad, USA). The program settings are as follows: 95 °C for 5 min, then 40 cycles of 95 °C for 15 s, 60 °C for 15 s, 72 °C for 15 s. *TaActin* was used as an internal reference gene to normalize the expression levels of the investigated DECs and miRNAs. SPSS 21.0 software was used to analyze the statistical significance of the data.

## Results

### Root phenotypes of different wheat varieties

**Fig. 1:**
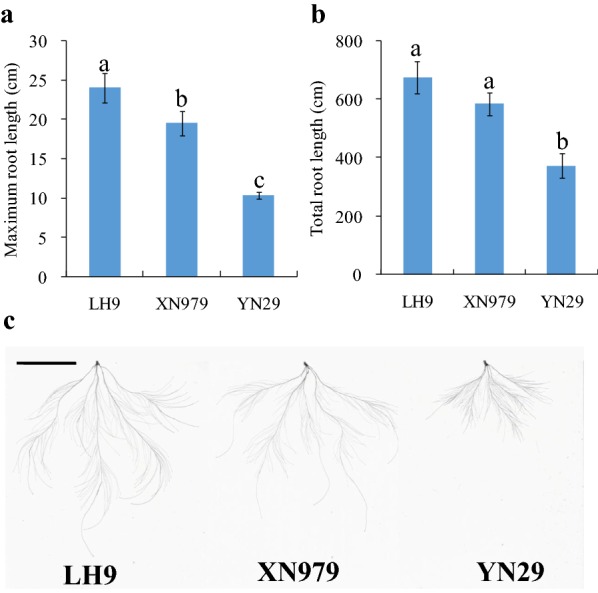
The root length and morphology of LH9, XN979 and YN29. The maximum root length (**a**), total root length (**b**) and root morphology (**c**) of LH9, XN979 and YN29. Bar = 5 cm

The root phenotypes of LH9, XN979 and YN29 are shown in Fig. 1. There are significant differences between the long root varieties (LH9, XN979) and the short root variety (YN29). The MRL of LH9 and XN979 were 13.7 cm and 9.2 cm longer than that of YN29 (Fig. 1a), and the TRL of LH9 and XN979 were 302.4 cm and 211.9 cm longer than that of YN29 respectively (Fig. 1b). Overall, the roots of LH9 and XN979 were significantly larger and deeper than those of YN29 (Fig. 1c).

### CircRNAs identification

**Fig. 2:**
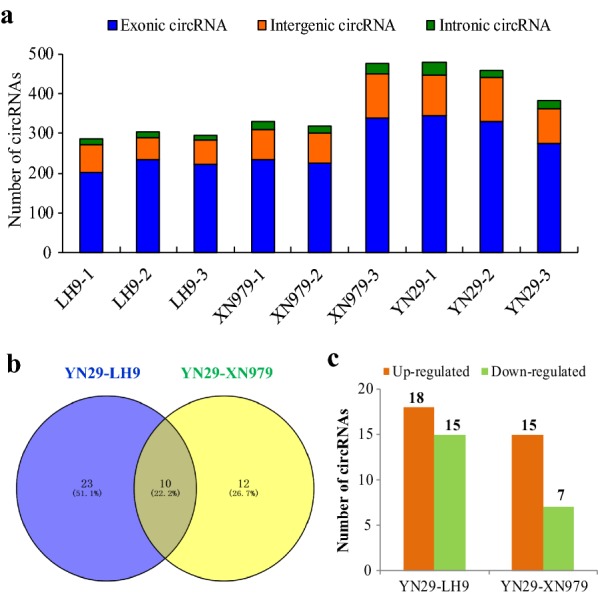
Statistical analysis of identified circRNAs and differentially expressed circRNAs (DECs) in the roots of LH9, XN979 and YN29. **a** The number of exonic circRNAs, intergenic circRNAs, and intronic circRNAs in each sequenced sample. **b** Venn diagram analysis of DECs in the YN29-LH9 and YN29-XN979 comparisons. **c** The number of up-regulated and down-regulated DECs in each comparison

To determine circRNAs involved in the regulation of root length, nine cDNA libraries from wheat roots of LH9, XN979 and YN29 were constructed and sequenced. Over 100 million raw reads were generated in each library (among 100.9 to 104.7 million raw reads in each library). About 76% to 85% reads were successfully mapped to wheat genome (TGAC v1.0). Find_circ software was employed to detect head-to-tail splicing (back-spliced) of the remaining 15–24% unmapped RNA-seq reads. The number of circRNAs identified in each sample ranged from 285 to 478. Among them, more than 70% of circRNAs are exonic circRNAs (Fig. 2a). The proportion of intronic circRNAs is the lowest, no more than 6.5% in each sample. The proportion of intergenic circRNAs is between the two, accounting for 18.7–24.9% (Fig. 2a).

### Identification of differentially expressed circRNAs

We compared the circRNAs expression profiles in the long root varieties (XN979 and LH9) and the short root variety (YN29), and obtained DECs between them. In the YN29-LH9 comparison, 33 circRNAs were identified as DECs (p value ≤ 0.05 along with |log_2_ (foldchange)| ≥ 1) (Fig. 2b; Additional file 2: Table S2). Among them, 18 circRNAs were up-regulated and 15 were down-regulated in YN29 compared with LH9 (Fig. 2c). We totally identified 22 DECs in the one to one comparison between YN29 and XN979 (Fig. 2b; Additional file 3: Table S3). Among them, 15 circRNAs showed up-regulation and seven circRNAs exhibited down-regulation in YN29 compared with XN979 (Fig. 2c). Among the circRNAs identified above, ten circRNAs existed in both the YN29-LH9 comparison and the YN29-XN979 comparison (Fig. 2b; Table 1). Twenty-three DECs were specifically found in the YN29-LH9 comparison and 12 DECs were specifically found in the YN29-XN979 comparison (Fig. 2b). Since the roots of both LH9 and XN979 are significantly larger and deeper than those of YN29 (Fig. 1c), the ten DECs coexisting in the two comparisons are highly likely to be involved in the regulation of wheat root length.

**Table 1:** CircRNAs involved in the regulation of wheat root length

Name	Position	Chr	Corresponding miRNAs
circRNA23	1B:60935-76949	TGACv1_scaffold_049914_1BS	tae-miR1134; tae-miR5085
circRNA2473	6B:456-24596	TGACv1_scaffold_500983_6BL	tae-miR164; tae-miR1134; tae-miR1122b-3p; tae-miR1125; tae-miR1118; tae-miR1137a; tae-miR1122a; tae-miR1133; tae-miR9773
circRNA3645	U:124270-137099	TGACv1_scaffold_641408_U	tae-miR1133; tae-miR1137a; tae-miR9773
circRNA1237	1A:102008-120691	TGACv1_scaffold_000490_1AL	None
circRNA3004	1B:69161-71982	TGACv1_scaffold_049372_1BS	None
circRNA2496	1D:61364-61592	TGACv1_scaffold_080514_1DS	None
circRNA3197	2B:52297-53454	TGACv1_scaffold_147474_2BS	None
circRNA832	2D:14143-15315	TGACv1_scaffold_178493_2DS	None
circRNA198	3A:8606-9403	TGACv1_scaffold_197368_3AL	None
circRNA956	7A:20092-74718	TGACv1_scaffold_569274_7AS	None

### Real-time PCR verification of DECs

**Fig. 3:**
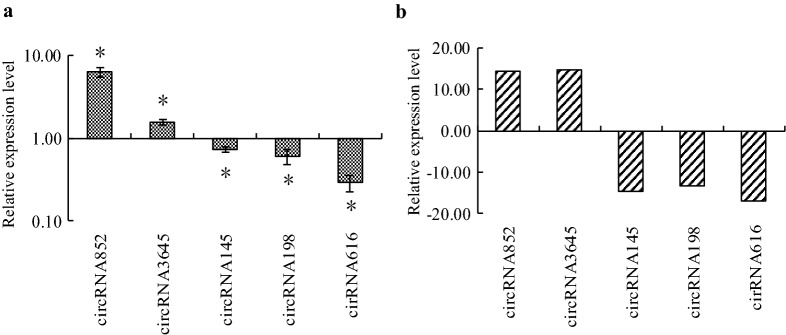
Relative expression analysis of differentially expressed circRNAs (DECs). **a** The results of relative expression analysis of circRNAs by using real-time PCR technology (*p < 0.05). **b** The results of relative expression analysis of circRNAs by using RNA-sequence technology

Five randomly selected DECs were selected for data verification of RNA-seq by using real-time PCR technology. Results showed that the expression levels of the five DECs detected using real-time PCR technology matched well with the results of RNA-seq (Fig. 3), indicating that the results of RNA-seq are reliable.

### The regulation of miRNAs levels by circRNAs

**Fig. 4:**
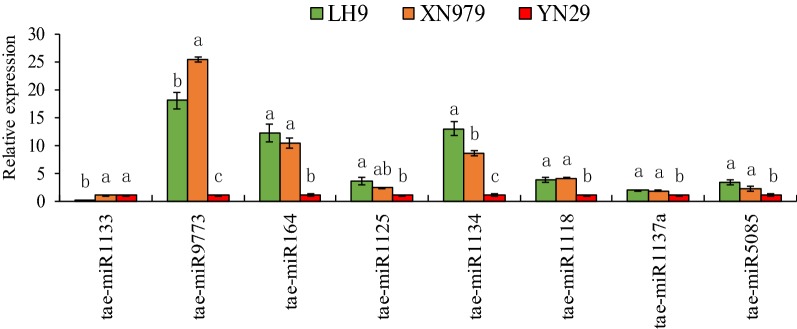
Relative expression analysis of potential binding miRNAs of the DECs in the roots of LH9, XN979 and YN29. Significant difference at p < 0.05 is indicated by different letters above the columns

It has been reported that circRNAs can act as ceRNAs (competitive endogenous RNA) of miRNAs, inhibiting their binding to mRNA molecules, thereby regulating gene expression [21, 44]. To detect whether the ten DECs coexisted in both comparisons can perform the function, psRNATarget software was employed to predict potential miRNA binding sites of the circRNAs. Results showed that three out of the ten DECs were predicted to have two to nine corresponding miRNAs binding sites (Table 1). CircRNA23 have putative miRNA binding sites for tae-miR1134 and tae-miR5085. CircRNA3645 have putative miRNA binding sites for tae-miR1133, tae-miR1137a and tae-miR9773. According to the psRNATarget-based prediction, circRNA2473 can bind to nine different miRNAs. To investigate whether these circRNAs regulate the expression of the corresponding miRNAs, eight potential circRNA-binding miRNAs were selected to check their expression levels in the long root plants (LH9 and XN979) and the short root plants (YN29). Results showed that six of them exhibited significant differences between the long root plants (LH9 and XN979) and the short root plants (YN29) (Fig. 4).

## Discussion

Deeper rooting is beneficial for plant production and survival under water and nitrogen deficient conditions [48–50]. Therefore, genetic improvement of root traits is essential for improving crop nutrient and water use efficiencies. Recent studies have shown that circRNAs are widespread in plants and play important roles in regulating plant growth and development, and stress tolerance [23–41]. However, whether circRNAs, as a new type of non-coding RNA molecules, participate in the regulation of root growth is not clear. Here, an integrated comparative root transcriptomic study of long root plants (LH9 and XN979) and short root plants (YN29) was conducted to explore this question. The number of circRNAs identified in each sample ranged from 285 to 478. More than 70% circRNAs belong to exonic circRNAs category. The intronic circRNAs only account for 3.9–6.5% of the total number of circRNAs in different samples (Fig. 2a). The proportion of intronic circRNAs is similar to the results of previous reports in *Arabidopsis* (3.8% intronic circRNAs), tomato (3.6%) and wheat (less than 6.5%) [16, 19, 41]. In the YN29-LH9 comparison and YN29-XN979 comparison, 33 and 22 DECs were identified, respectively (Fig. 2b). In total, ten circRNAs existed in both the YN29-LH9 comparison and the YN29-XN979 comparison. As has been mentioned above, both the roots of LH9 and XN979 were significantly larger and deeper than those of YN29 (Fig. 1c), therefore, the ten coexisted DECs in both comparisons should play more critical roles in the regulation of root length.

Previous studies in animal and human have confirmed that circRNAs can act as miRNA sponges to capture miRNAs from their target genes via ceRNA (competing endogenous RNAs) networks [51, 52]. Similar reports have recently been made in plants [26, 34, 38, 41]. To reveal whether the ten DECs can target miRNAs and participate in transcriptional regulation of genes, psRNATarget software was used to identify potential miRNA binding sites. As expected, three of the ten circRNAs had two to nine putative miRNA-binding sites (Table 1), which was consist with another study in wheat [38]. Moreover, the results of real-time PCR analysis showed that the expression levels of six out of the eight investigated potential binding miRNAs exhibited significant differences between the long root plants (LH9 and XN979) and the short root plants (YN29) (Fig. 4). Therefore, the expression levels of these miRNAs were correlated with their corresponding circRNAs. These results indicate that the three circRNAs may bind to the target miRNAs and modulate the transcript levels of these miRNAs. Interestingly, it has been reported that some putative target miRNAs are involved in the regulation of plant growth and development [53–57]. For example, miR164 has been reported controlling root development in *Arabidopsis*, maize and potato [53–55]. In *Arabidopsis,* one of miR164 target genes is *NAC1*, which transduces auxin signals for lateral root emergence [58, 59]. In *mir164* mutants, plants express less miR164 and more *NAC1* mRNA, and produced more lateral roots. Moreover, the mutant phenotypes can be complemented by expression miR164a and miR164b genomic sequences [53]. Besides, the target genes of the potential circRNA binding miRNAs such as miR1122, miR1125, miR1134 and miR1133 are also involved in plant growth, development, metabolism and stress response [56, 57]. Therefore, these circRNAs may regulate wheat root length by modulating target miRNAs levels. We also noticed that different circRNAs may have the same miRNA binding site, and one circRNA may have several corresponding miRNA binding sites (Table 1). Since the mechanism of how circRNAs regulate their target miRNA are not clear in plants so far and these miRNA binding sites are predicted by psRNATarget software, whether circRNA can bind to each of them still requires further experimental validation. Furthermore, how do the circRNAs that do not have miRNAs binding sites work? What are the mechanisms by which different circRNAs coordinately regulate the length of wheat roots? There is still a lot of work to be done to clarify the regulatory roles of these circRNAs.

## Conclusions

Our study revealed that the expression levels of some circRNAs in roots exhibited significant differences between the long root plants (LH9 and XN979) and the short root plants (YN29). Ten DECs involved in the regulation of wheat root length were successfully identified, three of which have potential miRNAs binding sites. The expression levels of putative circRNA-binding miRNAs were correlated with their corresponding circRNAs. These results provide new clues for investigating the functions of circRNAs in the regulation of wheat root length.

## Additional files

**Additional file 1: Table S1.** Primers used for real-time PCR.

**Additional file 2: Table S2.** List of differentially expressed circRNAs (DECs) in the YN29-LH9 comparison.

**Additional file 3: Table S3.** List of differentially expressed circRNAs (DECs) in the YN29-XN979 comparison.

## Abbreviations

BR: brassinosteroid
ceRNA: competing endogenous RNA
circRNAs: circular RNAs
DECs: differentially expressed circRNAs
MRL: maximum root length
rRNA: ribosomal RNAs
TRL: total root length
